# Endophytic bacterial diversity in the phyllosphere of Amazon *Paullinia cupana* associated with asymptomatic and symptomatic anthracnose

**DOI:** 10.1186/s40064-015-1037-0

**Published:** 2015-06-13

**Authors:** Andréa Cristina Bogas, Almir José Ferreira, Welington Luiz Araújo, Spartaco Astolfi-Filho, Elliot Watanabe Kitajima, Paulo Teixeira Lacava, João Lúcio Azevedo

**Affiliations:** Department of Genetics, “Luiz de Queiroz” College of Agriculture, University of São Paulo, Av. Pádua Dias 11, PO BOX 83, Piracicaba, SP 13400-970 Brazil; Department of Microbiology, Institute of Biomedical Sciences, University of São Paulo, Av. Prof. Lineu Prestes, 1374-Ed. Biomédicas II, Cidade Universitária, São Paulo, SP 05508-900 Brazil; Molecular Diagnostic Laboratory, Biotechnology Division, Federal University of Amazon, Av. Gal. Rodrigo Octávio Jordão, 3000, Manaus, AM 69.077-000 Brazil; Department of Plant Pathology and Nematology, ‘‘Luiz de Queiroz’’ College of Agriculture, University of São Paulo, Av. Pádua Dias 11, Piracicaba, SP 13418-900 Brazil; Center of Biological Sciences and Health, Federal University of São Carlos, Via Washington Luís km 235, PO BOX 676, São Carlos, SP 13565-905 Brazil

**Keywords:** *Colletotrichum*, Culture dependent, Endophytes, PCR-DGGE, Clone library, Microbial diversity

## Abstract

Endophytes colonize an ecological niche similar to that of phytopathogens, which make them candidate for disease suppression. Anthracnose is a disease caused by *Colletotrichum* spp., a phytopathogen that can infect guarana (*Paullinia cupana*), an important commercial crop in the Brazilian Amazon. We investigated the diversity of endophytic bacteria inhabiting the phyllosphere of asymptomatic and symptomatic anthracnose guarana plants. The PCR-denaturation gradient gel electrophoresis (PCR-DGGE) fingerprints revealed differences in the structure of the evaluated communities. Detailed analysis of endophytic bacteria composition using culture-dependent and 16S rRNA clone libraries revealed the presence of *Firmicutes*, *Proteobacteria*, *Actinobacteria*, *Bacteroidetes*, and *Acidobacteria* phyla*. Firmicutes* comprised the majority of isolates in asymptomatic plants (2.40E^−4^). However, cloning and sequencing of 16S rRNA revealed differences at the genus level for *Neisseria* (1.4E^−4^), *Haemophilus* (2.1E^−3^) and *Arsenophonus* (3.6E^−5^) in asymptomatic plants, *Aquicella* (3.5E^−3^) in symptomatic anthracnose plants, and *Pseudomonas* (1.1E^−3^), which was mainly identified in asymptomatic plants. In cross-comparisons of the endophytic bacterial communities as a whole, symptomatic anthracnose plants contained higher diversity, as reflected in the Shannon–Weaver and Simpson indices estimation (*P* < 0.05). Similarly, comparisons using LIBSHUFF and heatmap analysis for the relative abundance of operational taxonomic units (OTUs) showed differences between endophytic bacterial communities. These data are in agreement with the NMSD and ANOSIM analysis of DGGE profiles. Our results suggest that anthracnose can restructure endophytic bacterial communities by selecting certain strains in the phyllosphere of *P. cupana*. The understanding of these interactions is important for the development of strategies of biocontrol for *Colletotrichum*.

**Electronic supplementary material:**

The online version of this article (doi:10.1186/s40064-015-1037-0) contains supplementary material, which is available to authorized users.

## Background

Globally, Brazil is the unique commercial-scale producers of guarana [*Paullinia cupana* var. *sorbilis* (Mart.) Ducke]. It is estimated that at least 70% of the national market is intended for the manufacture of soft drinks, while the remainder is marketed in the forms of syrup, stick, powder, extract and other products (Bentes and Costa Neto 2011). Furthermore, the derivatives from guarana are economically valuable resources throughout the pharmaceutical and cosmetics industries (Kuri 2008) and are widespread in the global market. A study of the guarana fruits transcriptome performed by Ângelo et al. (2008) revealed the presence of secondary metabolites in this plant, such as flavonoids, which are powerful anti-oxidants, and the common stimulant caffeine; this study permitted a better elucidation of the biological properties of guarana extracts.

Santa Helena plantation is located in Maués, in the Central Amazonas region of Brazil and holds the largest genetic database for guarana with over 70.000 cultivated plants (AMBEV 2011). However, the production of guarana has been declining in the Amazon State and is actually low compared with that of Bahia State (IBGE 2013). The main factor limiting the production and expansion in the Amazon State is anthracnose, caused by *Colletotrichum* spp., which is considered the most serious disease of the guarana culture (Bentes and Barreto 2004). Anthracnose causes severe necrosis of young leaves and affects the plant in all growth stages, leading to total drying and decline of the guarana trees in severe cases (Trindade and Poltronieri 1997) and reducing up to 88% of crop production under traditional cultivation conditions (Araújo et al. 2002a).

Integrated control (the use of resistant clones, culture management and chemical control) has been recommended to prevent anthracnose and reduce production losses. However, there is no effective control of anthracnose disease caused by *Colletotrichum* in guarana plants (Bentes and Matsuoka 2002; Tavares et al. 2005). Because plants obtained by clonal multiplication have the same genotype and only a portion of them develop anthracnose, a possible explanation for the lack of symptoms may lie in the nature of the microbial community associated with these plants.

Endophytes are defined as microorganisms that inhabit the inner organs and tissues of a plant for at least one period of their life-cycle, without causing visible harm to the host (Azevedo et al. 2000). They can colonize an ecological niche similar to that of phytopathogens and can play an important role in protecting their host against pathogens (Lacava et al. 2006; Mejía et al. 2008; Rajendran et al. 2011). The biocontrol activity of these microorganisms may be due to niche competition (Lacava et al. 2004) and/or through synthesis of allelochemicals including antibiotics, lytic enzymes and siderophores (Araújo et al. 2001; Sturz and Christie 2003). In addition, endophytes can induce systemic resistance in the host plant (Gao et al. 2010). The presence of endophytes has been reported in all host plants (Rosenblueth and Martínez-Romero 2006).

Studies regarding endophytic bacteria and their community structure have been performed using culture-dependent approaches (Gagne-Bourgue et al. 2013; Xiong et al. 2013; Ji et al. 2014). Nevertheless, in recent years, culture-independent methods associated with cloning and sequencing have provided additional information on whole bacterial endophytic communities and have revealed changes in the structure and species composition due to the presence of abiotic (Peñuelas et al. 2011; Ma et al. 2013) and biotic factors such as the presence of pathogens (Lian et al. 2008; Trivedi et al. 2010). Therefore, the aim of this study was to assess the diversity and composition of endophytic bacterial communities in the phyllosphere of asymptomatic and symptomatic anthracnose Amazon *P. cupana* plants. We employed culture-dependent based plating and culture-independent methods involving 16S ribosomal RNA PCR-denaturation gradient gel electrophoresis (PCR-DGGE) and clone libraries of 16S rRNA. To our knowledge, this is the first report of the endophytic bacterial communities that colonize the phyllosphere of *P. cupana* plants and provides information regarding the association between anthracnose symptoms and endophytic bacteria.

## Methods

### Plant material

Leaf samples were randomly collected from asymptomatic (n = 5) and symptomatic (n = 5) anthracnose *P. cupana* trees (clone BRS-Maués 800) (n = 5 leaves for each plant).The collection was conducted in November 2010 in the AmBev´s Santa Helena Plantation, Maués/AM/Brazil, (3°15′10.2″S, 57°44′16.3″W). After collection, the samples were brought to the lab and processed.

### Sample processing

The leaves collected from each plant were washed individually with tap water and subsequently subjected to a surface-disinfection process by stepwise washing in 70% ethanol for 1 min, a sodium hypochlorite solution (2% available Cl^−^) for 2 min, and two rinses in sterilized distilled water (Araújo et al. 2001). To confirm the disinfection process, aliquots of the last sterile-distilled water wash were plated onto 10% trypticase soy agar (TSA-Merck, Sigma-Aldrich, USA) supplemented with 50 µg ml^−1^ benomyl. The plates were examined for bacterial growth after incubation at 28°C for 7 days.

### Isolation of endophytic bacteria

One gram of each disinfected leaf sample was aseptically cut, triturated in 5 ml of sterile phosphate-buffered saline (gl^−1^) NaCl, 8; Na_2_HPO4, 1.44; KH_2_PO4, 0.24; KCl, 0.20; pH 7.4) and incubated at 28°C under continuous agitation. Appropriate dilutions were plated onto 10% trypticase soy agar (TSA-Merck) supplemented with benomyl (50 µg ml^−1^), and the plates were incubated at 28°C for 7 days. After culturing, the colonies were purified and stored in a 70% glycerol solution at −80°C. The data analysis was performed with the SAS software package (SAS Institute Inc., Cary, NC, USA) using a completely randomized analysis. The bacterial counts were transformed using Log_10_ (CFU + 1) before implementing the ANOVA. Tukey´s test was used for further comparison of the means (*P* < 0.05). The bacterial suspensions obtained in this step were subsequently used for total DNA extraction.

### Amplification of the 16S rRNA gene and molecular identification of the isolates

The 16S rRNA gene partial sequence was amplified using colony PCR. The bacterial colonies were harvested from 10% trypticase soy agar (TSA-Merck), placed in microtubes containing 80 µl of sterilized distilled water and incubated 15 min at 90°C. Two microliters of bacterial suspension were used for the DNA source in the PCR reaction. The PCR was conducted in 50 µl containing 10 × buffer (10 mM KCl, 10 mM Tris–HCl, and pH 8.3) (Fermentas Life Sciences, Brazil), 0.2 mM dNTP, 3.75 mM MgCl_2_, 2.5 U *Taq* DNA polymerase (Fermentas Life Sciences, Brazil) and 0.2 µM forward P027F (5′GAGAGTTTGATCCTGGCTCAG 3′) primer (Heuer and Smalla 1997). The conditions for the amplification of 16S rRNA sequences consisted of an initial denaturation step of 94°C for 4 min, followed by 35 cycles of 94°C for 30 s, 62.5°C for 1 min, 72°C for 1 min and a final extension of 10 min at 72°C. The PCR products were analyzed by electrophoresis using 1% (w/v) agarose gel stained with ethidium bromide. The 16S rRNA gene PCR fragments were purified with polyethylene glycol (PEG) (20% PEG 8000; 2.5 mM NaCl) and sequenced at the Human Genome Research Center (HGRC) (Institute of Biosciences, University of São Paulo, São Paulo, SP, Brazil).

The 16S rRNA gene sequences obtained were compared with sequences available in the Ribosomal Database Project (http://rdp.cme.msu.edu) and classified using the RDP Classifier tool (http://rdp.cme.msu.edu/classifier/classifier.jsp). Significant differences among taxonomic groups in asymptomatic and symptomatic anthracnose samples were checked using the Lib Compare tool (http://rdp.cme.msu.edu/comparison/comp.jsp).

### Extraction of total DNA from plant samples

Five hundred milliliters of each leaf extract that contained endophytic bacteria (suspensions obtained from one gram of surface-disinfected leaf samples, as described above) were used for total DNA extraction using the MoBio Power Soil DNA extraction kit (MoBio Laboratories, Carlsbad, CA, USA) according to the manufacturer’s recommendations. The total DNA was visualized by electrophoresis on a 1% (w/v) agarose gel stained with ethidium bromide.

### PCR-DGGE analysis

The first PCR was conducted with primers 799 F (5′ AAC MGG ATT AGA TAC CCK G 3′) (Chelius and Triplett 2001) and 1492 R (5′ TAC GYT ACC TTG TTA CGA CT 3′). The PCR mixture (50 µl) consisted of 1 µl of total DNA obtained from plant samples, 10 × buffer (10 mM KCl, 10 mM Tris–HCl, pH 8.3 (Fermentas Life Sciences, Brazil), 0.4 µM each primer, 0.25 mM dNTP, 3.75 mM MgCl_2_, 1% (w/v) formamide and 2.5 U *Taq* DNA polymerase (Fermentas Life Sciences, Brazil). A PCR mixture without DNA was used as the negative control in all PCR experiments. The PCR reactions were performed in a PT-200 thermocycler (MJ Research, USA) programmed to 95°C for 3 min, followed by 35 cycles of 94°C for 20 s, 53°C for 40 s, 72°C for 40 s and a final extension of step of 7 min at 72°C. The nested PCR was performed using 1 µl of the PCR product from the first reaction and 0.4 µM primers U968┴CG and R1378 R (Heuer and Smalla 1997), with a denaturation step of 94°C for 4 min, followed by 35 cycles of 94°C for 1 min, 56°C for 1 min, 72°C for 1 min and a final extension of step of 10 min at 72°C. The PCR products (450 bp) were analyzed by electrophoresis in a 1% (w/v) agarose gel stained with ethidium bromide.

The PCR-DGGE analysis was based on the method described by Muyzer and Smalla 1998 and adapted by Araújo et al. (2002b) using an Ingeny PhorU apparatus (Ingeny, Goes, The Netherlands). The PCR products (473 bp) in equal amounts (about) were loaded onto 6% (w/v) polyacrylamide gels in 0.5 × TAE buffer (20 mM Tris–acetate, 0.5 mM EDTA, and pH 7.4). The polyacrylamide gels were prepared with denaturing gradients ranging from 35 to 65%, where 100% denaturant contained 7 M urea and 40% formamide. Electrophoresis was conducted at 100 V and 60°C for 15 h. The gel was stained with silver nitrate (Blum et al. 1987) and photographed under a white light transilluminator (VariQuest 100, FOTODYNE Incorporated, Hartland, WI, USA).

The DGGE profiles were analyzed with BioNumerics (Applied Maths NV). The images were normalized using markers, and matrices of the data sets, based on the presence or absence of bands, were generated using PRIMER 6 for Windows (PRIMER-E, Plymouth, United Kingdom). Similarity coefficients were calculated using the Bray-Curtis coefficient. The generated similarity matrices were used to construct nonmetric multidimensional scaling (NMDS) ordinations to observe patterns of similarity between samples, and the significance of these patterns was tested using ANOSIM statistics (Clarke and Green 1988). The calculation of similarity coefficients and ANOSIM statistics were conducted using PRIMER 6.

### Clone libraries construction

Ten clone libraries were generated from PCR products of the 16S rRNA gene. The PCR mixtures (50 µl) contained 1 µl template DNA (5–10 ng), 10 × buffer (10 mM KCl, 10 mM Tris–HCl, pH 8.3 (Fermentas Life Sciences, Brazil), 0.4 µM each primer 799 F (5′ AAC MGG ATT AGA TAC CCK G 3′) (Chelius and Triplett 2001) and 1492 R (5′ TAC GYT ACC TTG TTA CGA CT 3′), 0.2 mM dNTP, 3.75 mM MgCl_2_, 1% (w/v) BSA (bovine serum albumin) and 2.5 U *Taq* DNA polymerase (Fermentas Life Sciences, Brazil). These primers are specific to bacteria and have a low affinity for chloroplast DNA. The PCR amplification was performed with an initial denaturation step at 95°C for 3 min, followed by 25 cycles of 94°C for 20 s, 53°C for 40 s, 72°C for 40 s and a final extension of step of 7 min at 72°C. One microliter of the PCR product was used in a second reaction with the primers 968 F (without GC-clamp) and 1387 R in similar PCR mixture conditions and at a final volume of 50 µl. The nested PCR was conducted with an initial denaturation step of 94°C for 4 min, followed by 35 cycles of 94°C for 1 min, 62.5°C for 1 min, 72°C for 2 min and a final extension of 72°C for 10 min. The PCR mixture without DNA was used as a negative control in all PCR experiments.

PCR products of about 433 bp were purified using the GFX™ PCR DNA and Gel Band Purification Kit (GE Healthcare, UK). Ligation into pGEM®-T Easy Vector System II (Promega, Madison, WI, USA) and transformation into competent *Escherichia coli* DH5α cells (Promega, Madison, WI, USA) were performed according to the manufacturer’s instructions. The extracted plasmid DNA was subjected to sequencing with the 1378 R primer and BigDye® Terminator v 3.1 Cycle Sequencing Kit (Applied Biosystems, Foster City, CA, USA). The sequencing was performed in an ABI 3730 DNA Analyser (Applied Biosystems) at the Human Genome Research Center (HGRC) (Institute of Biosciences, University of São Paulo, São Paulo, SP, Brazil).

### Taxonomic assignment and phylogenetic analysis

The nucleotide sequences were analyzed for quality and trimmed using the CodonCode Aligner program (http://www.codoncode.com/aligner/). Only sequences with a quality parameter >20 (i.e., less than one error in 100 nucleotides) were considered (Ewing et al. 1998). Chimeras and chloroplast sequences were checked using Bellerophon v.3 on the Greengenes chimera-check tool (http://greengens.lbl.gov) and the MG-RAST metagenomics analysis server (http://metagenomics.anl.gov/metagenomics.cgi?page=Home), respectively. The phylogenetic affiliation was inferred by RDP classifier (http://rdp.cme.msu.edu/classifier/classifier.jsp).

A phylogenetic tree was constructed using Mega 4 software (Tamura et al. 2007). For this process, bacterial 16S rRNA gene sequences were chosen randomly based on classification of sequences representative of each genera and aligned with type sequences present in the RDP Project database (http://rdp.cme.msu.edu/index.jsp) using the Clustal W (http://www.ebi.ac.uk/Tools/msa/clustalw2/). The analyses were based on the Neighbor-Joining method following the Jukes–Cantor model for substitution of nucleotides. The robustness of the branches was tested using bootstrap analysis with 1,000 replications and the consensus tree edition was held using ITOL (http://itol.embl.de/).

Significant differences among taxonomic groups in asymptomatic and symptomatic anthracnose plants were checked using the Lib Compare tool (http://rdp.cme.msu.edu/comparison/comp.jsp).

### Richness estimation and diversity of total endophytic bacterial communities

Using MOTHUR v.1.20.3 software (http://www.mothur.org) (Schloss 2009), the sequences were aligned and the evolutionary distances were calculated using the Jukes–Cantor parameter (Jukes and Cantor 1969). The generated matrix was used to assign sequences to operational taxonomic units (OTUs) and to generate estimations of the Chao1 richness, Shannon–Weaver and Simpson diversity index (at 100, 97, 95 and 91% similarity levels), rarefaction curves and heatmaps. The LIBSHUFF software was used to determine the significant differences between asymptomatic and symptomatic anthracnose plants (Schloss et al. 2004).

### Nucleotide sequences accession numbers

The 16S rRNA gene sequences obtained from the isolation methodology and construction of clone libraries were deposited in the GenBank database with accession numbers KC493265 to KC493350 and KC348605 to KC349130, respectively.

## Results

### Determination of culturable endophytic bacteria

**Figure 1:**
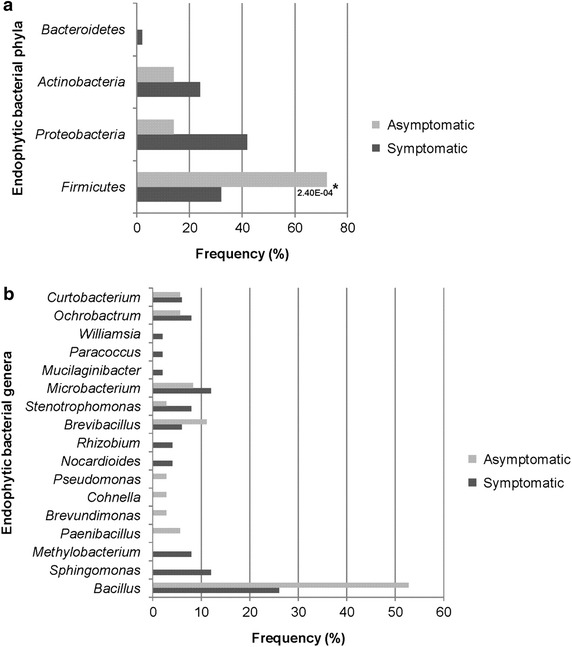
Relative frequency distribution of phyla (**a**) and genera (**b**) of endophytic bacteria isolated from asymptomatic and symptomatic anthracnose leaf samples of *P. cupana* (n = 86). A significant difference (*P < 0.05) was observed at the phylum level for *Firmicutes* in asymptomatic plants.

The diversity of culturable endophytic bacteria was assessed in samples of leaves from 10 *P. cupana* plants. The bacterial densities were different (*P* < 0.05) according to the phytosanitary condition and ranged from 10^4^ to 10^5^ CFU g^−1^ (fresh leaf weight) for asymptomatic and symptomatic anthracnose plants, respectively. A total of 86 sequences were examined on the basis of 16S rRNA gene sequencing. The endophytic isolates were assigned, in order, to the *Firmicutes* (48.8%), *Proteobacteria* (30.2%)*, Actinobacteria* (19.7%) and *Bacteroidetes* (1.16%) phyla. With the exception of the *Bacteroidetes*, which was observed only in symptomatic anthracnose plants, all of the other phyla were present in all of the plants. *Firmicutes* comprised the majority of the isolates and was predominant in asymptomatic plants (2.40E^−4^) (Figure 1a). The most abundant classes (30.2% of *Proteobacteria*) were *Gammaproteobacteria* (6.9%) and *Alphaproteobacteria* (23.3%).

The isolates could also be assigned to the genera *Bacillus* (37.2%), *Microbacterium* (10.5%), *Brevibacillus* (8.1%), *Sphingomonas* (6.9%), *Ochrobactrum* (6.9%), *Stenotrophomonas* (5.8%), *Curtobacterium* (5.8%), *Methylobacterium* (4.7%), *Paenibacillus* (2.3%), *Rhizobium* (2.3%), and *Nocardioides* (2.3%). Additionally, we found *Cohnella*, *Brevundimonas*, *Pseudomonas*, *Paracoccus*, *Williamsia* and *Mucilaginibacter*, but they represented only 1.2% of genera assessed. The presence, absence or prevalence of each genus also varied according to the physiological state of the plants (Figure 1b). No significant differences were observed among isolates at the genus level.

### PCR-DGGE analysis

**Figure 2:**
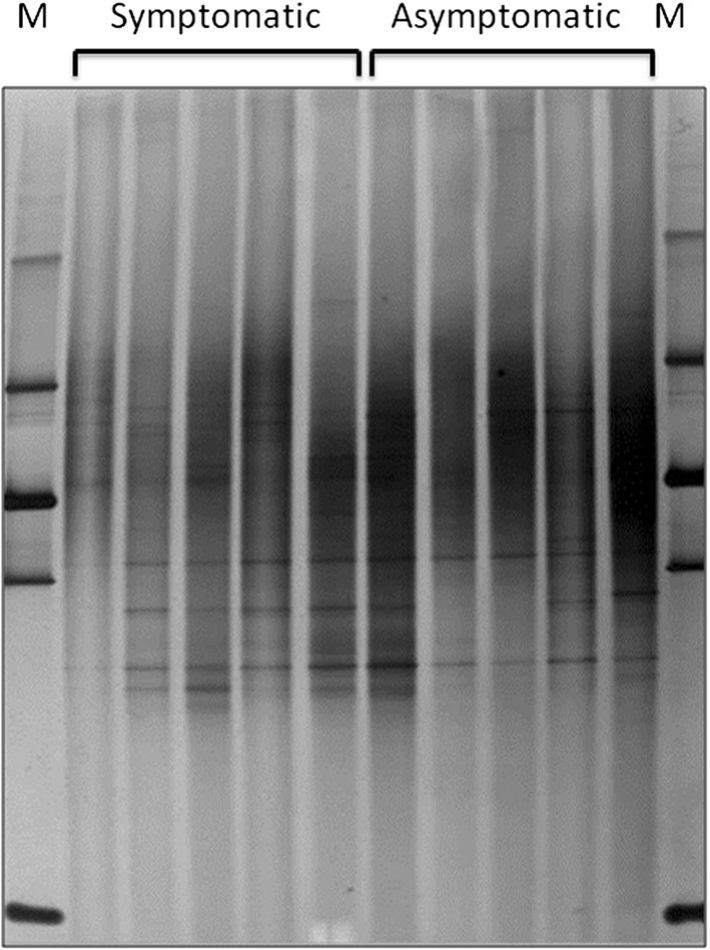
Denaturing gradient gel electrophoresis (DGGE) fingerprints of endophytic bacteria in symptomatic and asymptomatic anthracnose leaf extracts of *P. cupana*. Each line represents a repetition for each treatment. The image was normalized using marker (M −100 bp) and DDGE profiles were analyzed with Bionumerics software (Applied Maths NV).

**Figure 3:**
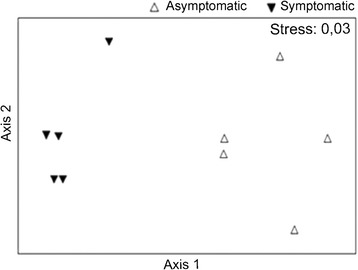
NMDS analysis comparing the endophytic bacterial community structure of asymptomatic and symptomatic anthracnose plants. *Each point* represents the DGGE profiles for each treatment.

The 16S rRNA gene-based PCR-DGGE analysis revealed the presence of endophytic bacteria in all leaf samples (Figure 2) NMDS analysis of the DGGE profiles showed that the structure of endophytic bacterial communities of asymptomatic and symptomatic anthracnose plants are different and suffered significant impact due to disease (ANOSIM R = 0.972, *P* < 0.001) (Figure 3).

## 16S rRNA clone library analysis

After examining the sequences for quality and the presence of chimeras and chloroplasts, a total of 526 clone sequences were selected, with 260 and 266 from symptomatic anthracnose and asymptomatic plants, respectively.

**Figure 4:**
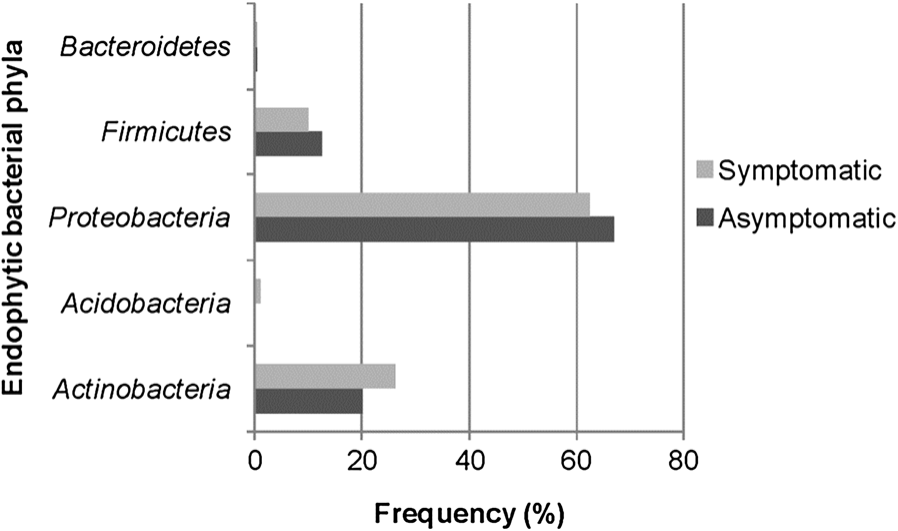
Composition of 16S rRNA gene clone libraries at the phylum level determined by similarity with RDP Classifier tool (n = 526).

Using the RDP classifier, *Proteobacteria* (70.65%), *Actinobacteria* (23.68%), *Firmicutes* (4.69%), *Acidobacteria* (0.79%) and *Bacteroidetes* (0.19%) were the dominant phyla. Clones representing *Bacteroidetes* were found only in the clone library of the asymptomatic plants, whereas the phylum *Acidobacteria* was represented only in symptomatic anthracnose plants (Figure 4). Among the sequences similar to *Proteobacteria*, 356 were associated with *Gammaproteobacteria* (54.7%), *Betaproteobacteria* (38.48%) and *Alphaproteobacteria* (6.74%).

**Figure 5:**
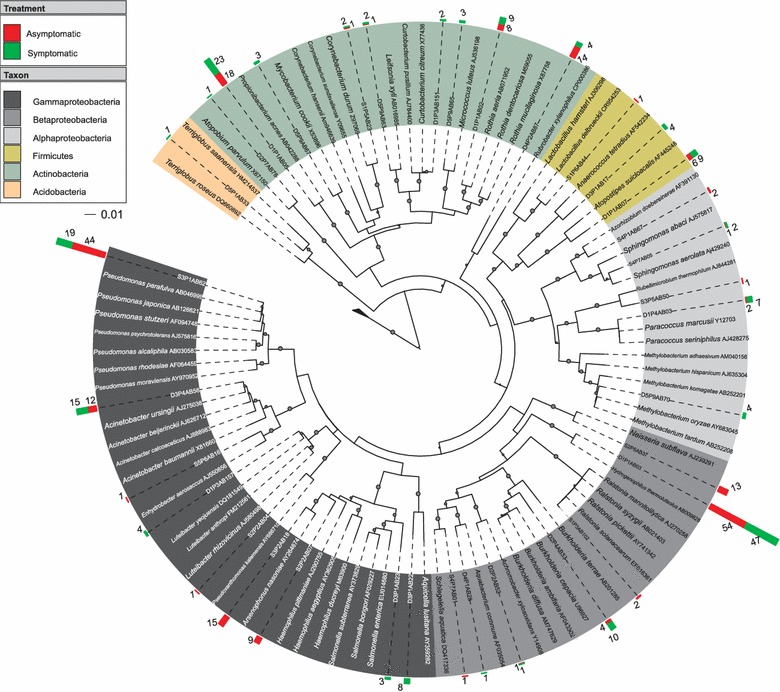
Phylogenetic analysis of 16S rRNA genes of endophytic bacterial communities present in asymptomatic and symptomatic anthracnose *P. cupana*. Bootstrap values (1,000 repetitions) above 50% are represented by *circles* in tree branches. The tree shows sequences representatives of the genera, which were randomly chosen from the similarity analysis performed by the RDP Classifier. The representative sequences were aligned only with “Type” sequences, and the proportion that each represents is represented by *colored bars* in proportional size and color corresponding to the treatment source (asymptomatic and symptomatic), in addition to the *number beside the bar*. The different taxonomic groups can be distinguished by the *color* of the leaves of the phylogenetic tree.

Furthermore, 397 sequences were classified to the genus level. Among these sequences, 34 were randomly chosen as representatives in the phenetic analysis. The distribution of the represented sequences is illustrated by a proportionally sized bar and color corresponding to the treatment of the origin (Figure 5). These sequences were aligned with “type” sequences obtained from the RDP database and affiliated with 34 bacterial genera, which were mainly represented by *Hydrogenophilus* (25.4%), *Pseudomonas* (15.8%), *Propionibacterium* (10.3%), *Acinetobacter* (6.8%), and *Rubrobacter* (4.5%), and other groups were present in minor proportions as *Rothia* (4.2%), *Arsenophonus* (3.7%), *Atopostipes* (3.7%), *Burkholderia* (3.5%), *Neisseria* (3.2%), *Haemophilus* (2.2%), *Paracoccus* (2.2%) and *Aquicella* (2%). Another 21 genera represented less than 1% of the total.

**Figure 6:**
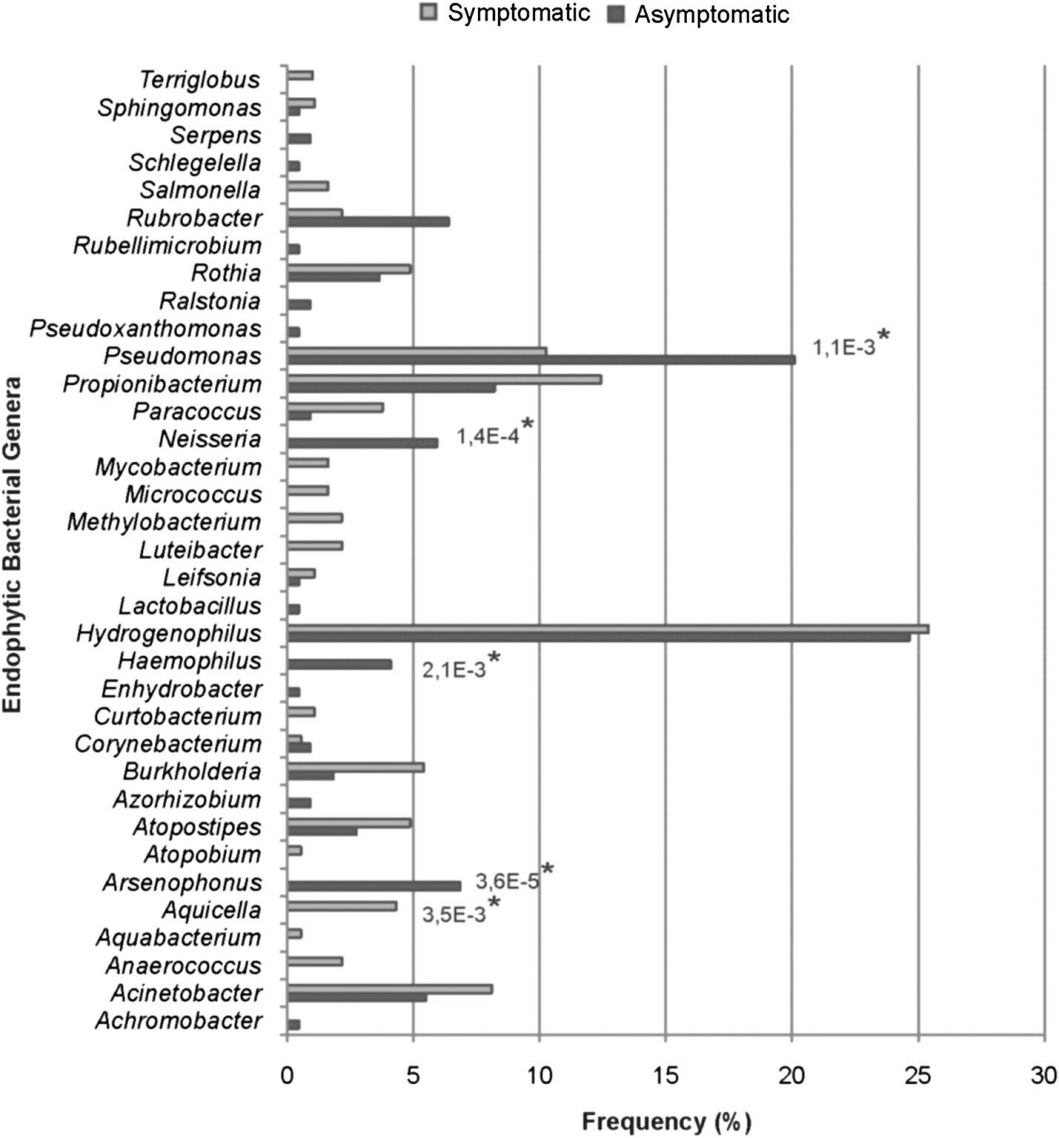
Comparisons of the 16S rRNA gene clone library from asymptomatic and symptomatic anthracnose *P. cupana* plants (n = 410). Significant differences (*P < 0.05) were observed among *Pseudomonas*, *Neisseria*, *Haemophilus*, *Arsenophonus* and *Aquicella* at the genera level.

The 16S rRNA gene clone libraries were also compared using the Lib Compare tool (Figure 6). Significant differences (*P* < 0.05) were observed at the genera level among *Neisseria* (1.4E^−4^), *Haemophilus* (2.1E^−3^) and *Arsenophonus* (3.6E^−5^) found only in asymptomatic plants, *Aquicella* (3.5E^−3^) found only in symptomatic anthracnose plants, and *Pseudomonas* (1.1E^−3^) was observed in both treatments but at the highest frequency in asymptomatic plants.

### Richness estimation and diversity of total endophytic bacterial communities

**Figure 7:**
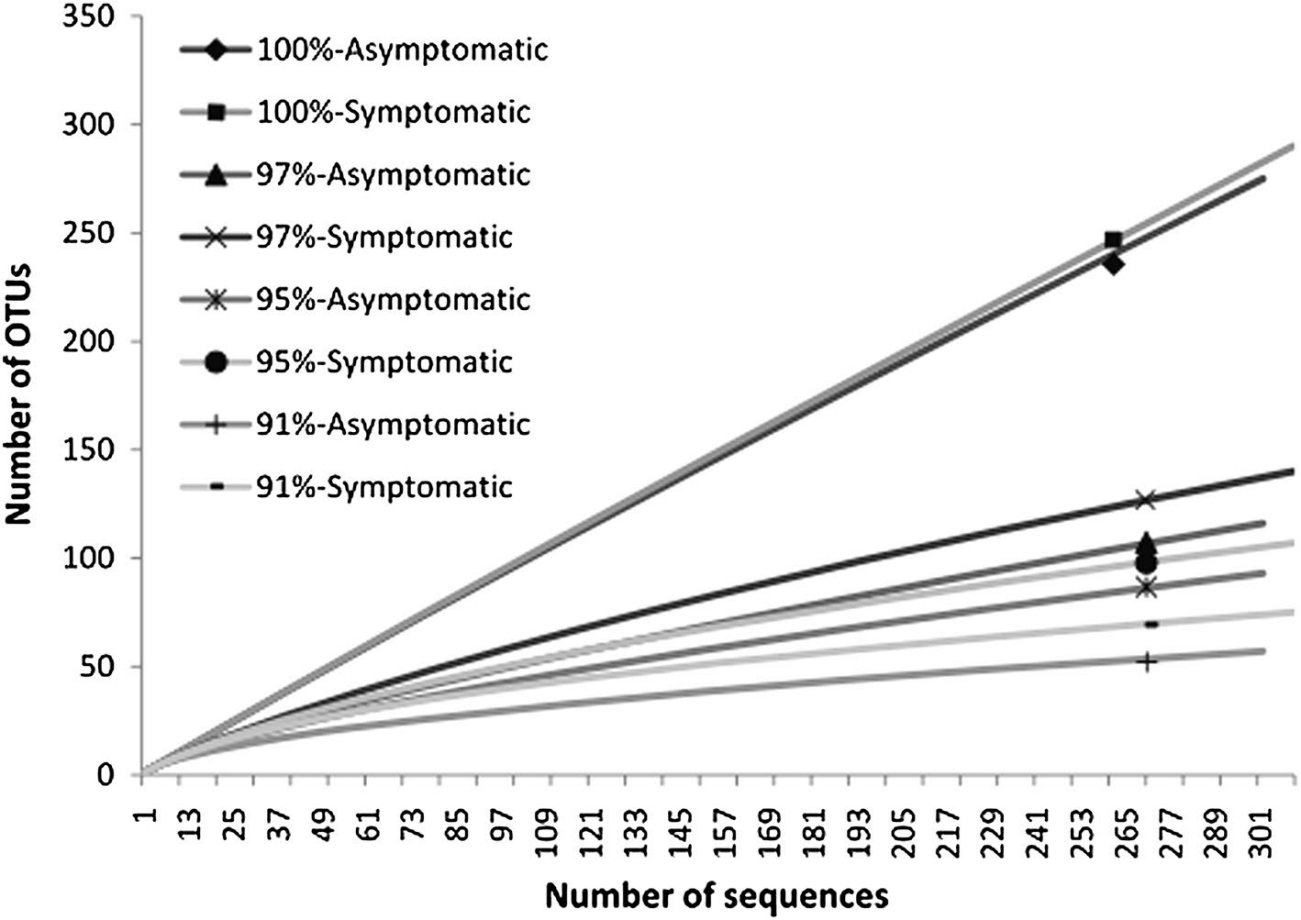
Rarefaction curves of the 16S rRNA gene of culturable and unculturable endophytic bacterial communities associated with asymptomatic and symptomatic anthracnose *P. cupana* leaves.

Rarefaction analyses were performed using cut-off criteria for grouping OTUs at species (97%), genus (95%) and order (91%) levels. The curves showed a tendency to stabilize at the 97% level for both asymptomatic and symptomatic anthracnose plants and reached a plateau at the 91% level of similarity (Figure 7). This indicates that the number of sequences analyzed is large enough to reflect the diversity of culturable and unculturable endophytic bacterial in *P. cupana.*

Using the same cut-off criteria for the grouping of OTUs employed for the construction of rarefaction curves, Chao1 richness and Shannon–Weaver (H) and Simpson diversity indices could also be determined. Chao1 richness estimations (*P* < 0.05) did not differ significantly between asymptomatic and symptomatic anthracnose plants. However, Shannon–Weaver (H) at the 97–91% levels and the Simpson index at the 91% level for symptomatic anthracnose plants were significantly higher than those observed for asymptomatic plants (Additional file 1: Table S1).

The analysis of richness using the Chao1 estimator indicated that both symptomatic and asymptomatic plants did not differ significantly. However, the Shannon–Weaver (H) and Simpson indices indicate that the diversity of the OTUs corresponding order (91%) is significantly higher in symptomatic plants. Moreover, the Shannon–Weaver (H) indices also indicated the highest species diversity (97%) in plants symptomatic for anthracnose.

**Figure 8:**
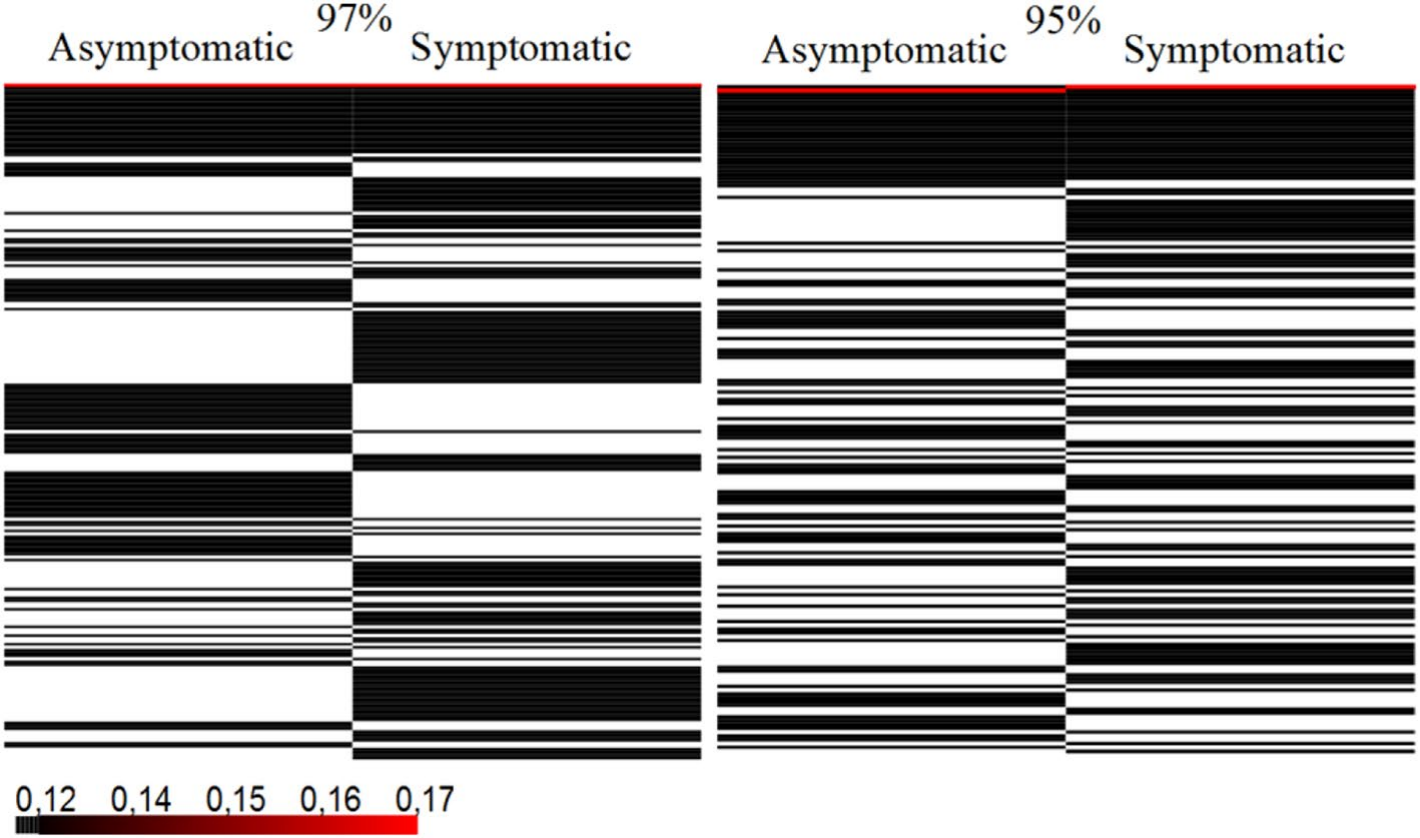
Heatmaps for the relative abundance of operational taxonomic units (OTUs) for the 16S rRNA gene of *P. cupana* leaf-associated endophytic bacteria (n = 612). The relative abundance for the OTUs at 97 and 95% levels of similarity is shown at the *left* and *right* side, respectively. *Side-by-side lines* represent OTUs shared by asymptomatic and symptomatic anthracnose plants. The OTUs with a low frequency of sequences (less than 0.14) are represented by *black lines*, while OTUs with a higher frequency of sequences (higher than 0.16) are represented by *red lines.*

Heatmaps for the relative abundance of OTUs were also generated (Figure 8). An analysis at the 97% level of similarity revealed the presence of 140 OTUs in symptomatic anthracnose and 116 OTUs in asymptomatic plants, with 10.34% of the OTUs shared between plants. When analyzed at 95%, 107 OTUs were observed on symptomatic anthracnose and 93 on asymptomatic plants, with 14.28% shared (Additional file 1: Table S1). A density analysis of groups within the OTUs revealed the presence of a more dense but non-shared group between the treatments at the 95% level of similarity.

A cross-comparison of asymptomatic and symptomatic anthracnose plants using LIBSHUFF statistics revealed that the endophytic bacterial community present in asymptomatic plants is significantly different from the symptomatic anthracnose (*P* < 0.0001). These data are in accordance with NMSD and ANOSIM analysis of DGGE profiles.

## Discussion

To investigate the endophytic bacterial communities living in the phyllosphere of asymptomatic and symptomatic anthracnose Amazon *P. cupana* plants, we used culture-dependent and culture-independent approaches. The application of both methods in parallel (for the same samples) for assessing bacterial communities in leaves has been previously reported (Araújo et al. 2002b; Ulrich et al. 2008; Yashiro et al. 2011) and is important in analyzing microbial diversity because the analysis based on the culture-dependent method may underestimate the diversity (Rasche et al. 2006; Jackson et al. 2013). In fact, we found fewer endophytic bacteria using the culture-dependent method than the 16S rRNA clone libraries. In addition to the accuracy of the methodologies, several abiotic and biotic environmental factors may affect the plant physiology and consequently the assessed microbial communities. In this context, the presence of pathogens has been considered an important factor in the restructuring of endophytic bacterial communities (Araújo et al. Araújo et al. 2002a, b; Bulgari et al. 2011; Trivedi et al. 2010), which can play an important role in host plant protection (Lacava et al. 2006; Mejía et al. 2008).

When we employed PCR-DGGE fingerprinting to analyze the overall diversity of endophytic bacterial communities in *P. cupana*¸ we verified that asymptomatic and symptomatic anthracnose plants differed significantly (ANOSIM, R = 0.972) in their structure. Thus, the presence of *Colletotrichum* in *P. cupana* seems to be important to cause shifts in the microbial communities. Trivedi et al. (2010) suggested that these shifts can occur by mechanisms such as competition for nutrients and space, microbial cross talk and changes in the niche environment.

Regarding the identity of the obtained isolates and clones, we verified the presence of *Proteobacteria*, *Firmicutes*, *Actinobacteria*, *Bacteroidetes* and *Acidobacteria* phyla, which are reported in a phyllosphere (Romero et al. 2014; Rastogi et al. 2012; Bodenhause et al. 2013).

Some differences were observed in relation to the frequency and distribution of phyla in the samples analyzed. The *Firmicutes* phylum (48.8%), represented mainly by *Bacillus* (37.2%), was significantly more isolated from asymptomatic plants (2.40E^−4^), followed by *Proteobacteria* (30.2%). This result differs from those observed in other studies, in which *Proteobacteria* has been isolated as the dominant phylum in the phyllosphere of other hosts (Costa et al. 2012). However, sequences affiliated with *Proteobacteria* (70.65%) represented the largest fraction of clones, and corroborated with studies that have reported this phylum as the most common in leaves when obtained by culture-independent approaches (Romero et al. 2014; Sagaram et al. 2009; Kim et al. 2012). Among the members of *Proteobacteria*, *Pseudomonas* was significantly assessed (1.1E^−3^) in asymptomatic plants.

The bacteria representing *Firmicutes* and *Proteobacteria* found in our study have also been observed in high frequency colonizing the leaves of other plants without disease symptoms (Bodenhause et al. 2013; Melnick et al. 2011; Paz et al. 2012). This finding could make *Bacillus* and *Pseudomonas* interesting biological control agents against phytopathogens by various mechanisms that may include induction of systemic resistance in the host plant and antibiosis (Choudhary and Johri 2009; Krid et al. 2010). In this context, we speculate that there is an association of these endophytic bacteria with the resistance of *P. cupana* to anthracnose, which could be limiting the invasion and the establishment of *Colletotrichum* in the asymptomatic plants.

Other clones representative of *Proteobacteria* significantly assessed in asymptomatic plants were *Neisseria* (1.4E^−4^), *Haemophilus* (2.1E^−3^) and *Arsenophonus* (3.6E^−5^). Although *Neisseria* is typically associated as a human pathogen, this genus has been found inhabiting the interior of plants (Videira et al. 2013). *Haemophilus*, also described as a human pathogen, was associated with the hyphae of endophytic fungi isolated from *Cupressus arizonica* (Hoffman and Arnold 2010). *Arsenophonus* genus, however, has been described as a group of insect intracellular symbionts (Nováková et al. 2009). To our knowledge, this is the first association of *Haemophilus* and *Arsenophonus* as endophyte of the phyllosphere.

In the clone libraries of symptomatic anthracnose plants, the *Aquicella* (3.5E^−3^) genus was significantly assessed. These bacteria are typically found in water samples (Perkins et al. 2009; Santos et al. 2003) but were recently found as endophytes in clone libraries of the root of *Pennisetum purpureum* (Videira et al. 2013). Disease abatement is possible using the establishment of some endophytes in the host plant or the allowing endophytes to trigger the disease by synergistic interaction with a pathogen (Araújo et al. 2002b). However, we cannot affirm whether this would occur in *P. cupana* plants colonized by *Aquicella.*

Although examined by isolation and cloning, the presence of *Actinobacteria* and *Bacteroidetes* phyla in both asymptomatic and symptomatic anthracnose plants was not significant in relation to the phytosanitary condition. *Actinobacteria* was the third most isolated phylum (19.7%) and the second most identified in the clone libraries (23.67%) and typically established associations with the phyllosphere of other hosts (Bodenhause et al. 2013; López-Velasco et al. 2011). *Bacteroidetes* was the least isolated phylum (1.16%) from symptomatic anthracnose plants and the least identified in the clone libraries of asymptomatic plants (0.19%). Costa et al. (2012) also isolated *Bacteroidetes* from the leaves of *Phaseolus vulgaris* at low frequency. In contrast, Jackson et al. (2013) reported this phylum as one of most prevalent in the phyllosphere of vegetables when identified by a culture-independent method.

*Acidobacteria* was the unique phylum that was not observed among isolates. Although typically found in the rhizosphere (Gottel et al. 2011; Bulgarelli et al. 2013), we identified this phylum at low frequency (0.79%), inhabiting the phyllosphere of symptomatic anthracnose *P. cupana*. Recently, *Acidobacteria* was also obtained as endophytes of leaves by pyrosequencing (Romero et al. 2014), which confirmed our data obtained by the culture-independent method.

*Alphaproteobacteria* (23.3%) was the most abundant class of *Proteobacteria* among isolates, followed by *Gammaproteobacteria* (6.9%). In contrast, a high number of *Gammaproteobacteria* (54.7%) was found in the clone libraries, followed by *Betaproteobacteria* (38.48%) and *Alphaproteobacteria* (6.74%). Similar to what we found in our research, *Alphaproteobacteria* and *Gammaproteobacteria* are described in some studies as dominant in the phyllosphere (Romero et al. 2014; Jin et al. 2013). *Betaproteobacteria* can form a considerable part of the bacterial communities in some situations (Jackson et al. 2013). These classes did not differ significantly in relation to the phytosanitary condition.

Our rarefaction analysis showed that the number of isolates and clones evaluated was a sufficient representation of the sampling effort of endophytic bacterial communities in *P. cupana*. Asymptomatic and symptomatic anthracnose plants did not differ in relation to their species richness. However, the Shannon–Weaver and Simpson index showed higher microbial diversity in symptomatic anthracnose plants. Heatmaps for the relative abundance of OTUs also revealed a higher density in symptomatic anthracnose plants. LIBSHUFF statistics confirmed these differences in the composition of endophytic bacteria between asymptomatic and symptomatic anthracnose plants.

Reiter et al. (2002) reported an increase in the diversity of endophytic bacteria in potato plants in response to *Erwinia carotovora* infection. Lian et al. (2008) also demonstrated that a tissue culture banana plantlet infected with *Fusarium oxysporum* had an increase in endophytic bacterial diversity. The infection of the host plant by some phytopathogens may involve the production of cell wall-degrading enzymes (Barras et al. 1994; Annis and Goodwin 1997), and the onset of lesions can allow the entry of other bacteria into the plant (Trivedi et al. 2010). In addition, the pathogen infection may affect plant physiology by favoring the establishment of some endophytic bacterial groups (Hallmann et al. 1998). In this context, the possible physiological changes in *P. cupana* due to anthracnose seem to be reflected in the distribution of endophytic bacteria between asymptomatic and symptomatic anthracnose plants.

For the first time, we have described the endophytic bacterial diversity in the phyllosphere of Amazon *P. cupana* plants. Our results suggest a possible interaction between anthracnose and the endophytic bacterial communities evaluated. Beneficial bacteria found in asymptomatic plants open the possibility for a better understanding of the mechanisms involved in the resistance of *P. cupana* to *Colletotrichum* spp. and the development of future strategies of biocontrol.

## Additional files

**Additional file 1: Table S1.** Number of OTUs, diversity and richness estimations for 16S r RNA gene.
