# Supplementary material for: Endophytic bacterial diversity in the phyllosphere of Amazon Paullinia cupana associated with asymptomatic and symptomatic anthracnose
Source: Springerplus. 2015 Jun 13;4:258. doi: 10.1186/s40064-015-1037-0 (PMC4467821; doi:10.1186/s40064-015-1037-0)
Supplement: Supplementary file 1 — Additional file 1: Table S1. Number of OTUs, diversity and richness estimations for 16S r RNA gene. [file 40064_2015_1037_MOESM1_ESM.docx]

TABLE S1

| **Similarity** | **Treatment** | **Number of sequences** | **OTUs** | **Shannon´s index (H´)** | **Simpson´s index**  **(D)** | **Chao1 estimator** |
| --- | --- | --- | --- | --- | --- | --- |
| 100% | Asymptomatic | 302 | 275 | 5,55 (5,46-5,64) | 0,0012 (0,0003-0,0021) | 3642 (2084-6543) |
|  | Symptomatic | 310 | 290 | 5,62 (5,54-5,71) | 0,0008 (0,0002-0,0013) | 6800 (3395-13940) |
| 97% | Asymptomatic | 302 | 116 | 3,79 (3,61-3,97)* | 0,0584 (0,0427-0,0741) | 417 (272-698) |
|  | Symptomatic | 310 | 140 | 4,17 (4,00-4,34)* | 0,0391 (0,0273-0,0508) | 398 (284-599) |
| 95% | Asymptomatic | 302 | 93 | 3,51 (3,33-3,69)* | 0,0695 (0,0527-0,0863) | 227 (160-362) |
|  | Symptomatic | 310 | 107 | 3,90 (3,73-4,06)* | 0,0435 (0,0317-0,0552) | 254 (181-399) |
| 91% | Asymptomatic | 302 | 57 | 3,01 (2,85-3,16)* | 0,0882 (0,0719-0,1045)* | 112 (79-195) |
|  | Symptomatic | 310 | 75 | 3,52 (3,38-3,67)* | 0,0527 (0,0410-0,0645)* | 166 (114-289) |

*(p<0,05)
